# Survivorship in Tumors of the Sinonasal Tract: The Need for Improved Awareness, Patient Education, and an Emphasis on Multi-Disciplinary Care

**DOI:** 10.3390/cancers17101666

**Published:** 2025-05-15

**Authors:** Jacklyn Liu, Anthony Tang, Umar Rehman, Marci L. Nilsen, Carl H. Snyderman, Nyall R. London, Valerie J. Lund, Matt Lechner

**Affiliations:** 1 Division of Surgery and Interventional Science, University College London, London E1 6AN, UK; 2 UCL Cancer Institute, University College London, London E1 6AN, UK; 3 Department of Otolaryngology, University of Pittsburgh School of Medicine, Pittsburgh, PA 15201, USA; 4 Department of Acute and Tertiary Care, School of Nursing, University of Pittsburgh, Pittsburgh, PA 15201, USA; 5 Department of Otolaryngology–Head and Neck Surgery, Johns Hopkins School of Medicine, Baltimore, MD 21201, USA; 6 Sinonasal and Skull Base Tumor Program, Head and Neck Surgery Branch, National Institute of Deafness and Other Communication Disorders, National Institutes of Health, Bethesda, MD 20810, USA; 7 Royal National Throat, Nose and Ear Hospital and Head and Neck Centre, University College London Hospitals NHS Trust, London E1 6AN, UK

**Keywords:** sinonasal cancer, survivorship, quality of life, long-term effects, late effects, financial toxicity

## Abstract

Sinonasal cancer treatment confers extensive and diverse sequela, which may persist for months to years after treatment or manifest as late effects. Furthermore, recurrences are common for some subtypes and may occur beyond five years post-treatment of the initial malignancy. Altogether, these can place a substantial physical, psychosocial, and economic burden on the survivor. Due to the rarity of these cancers, there are limited data to comprehensively elucidate the landscape of treatment-related morbidity in the long term. Furthermore, survivors may lack awareness of the entirety of possible adverse effects, which may exacerbate their long-term psychosocial well-being and quality of life, and delay attainment of appropriate care. To enable the development of patient education strategies and provide clinicians with up-to-date, evidence-based information on the long-term and/or late morbidity associated with sinonasal cancer treatment, a comprehensive review was performed. There is a wide range of issues that survivors face, both due to the sinonasal cancer itself and as a result of the treatment, highlighting the need for multidisciplinary survivorship care. Importantly, survivorship care will greatly benefit from patient and public involvement, alongside input from medical, surgical, and allied health professionals, to ensure that all aspects of care are addressed throughout the survivor journey.

## Simple Summary

Treatment for cancers of the sinuses and nose can lead to a wide range of long-term health problems, some lasting years or appearing much later. Recurrence of the cancer is also common, even after five years. These challenges can take a major toll on survivors physically, emotionally, and financially. As the cancer is rare, there is not much research on the long-term effects of treatment, and many survivors are not fully aware of what to expect. To address this, the authors reviewed existing studies to better understand these long-term effects. Their findings highlight the need for comprehensive, multi-disciplinary care for survivors and stress the importance of involving patients and the public in planning this care.

## 1. Introduction

Sinonasal cancers are a heterogenous and rare group of tumors which arise in the nasal cavity and/or paranasal sinuses and often involve adjacent critical structures, such as the orbit, skull base, and cranial nerves. Presentation typically involves non-specific sinonasal symptoms, including nasal obstruction and/or epistaxis/serosanguinous nasal discharge, which overlap with those of other benign nasal pathologies. Consequently, these tumors are often diagnosed at a later, more advanced stage [1].

There is an increasing number of sinonasal histologies owing to crucial differences in the underlying biological mechanisms, which gives rise to wide variation in the behavior and clinical course of these tumors [2,3,4,5]. As such, prognosis and course of treatment are highly dependent on histology [6,7,8,9,10]. The most common sinonasal cancer is squamous-cell carcinoma (SNSCC), accounting for 61% of cases [1,10]. Other types include intestinal-type adenocarcinoma (ITAC) [1,4,10], which represents 4.6%, olfactory neuroblastoma (ONB), comprising 2–6% [1,6,10], and adenoid cystic carcinoma (ACC), which accounts for 5–15% of all sinonasal cancers [1,9,10]. Less frequently, sinonasal mucosal melanoma (SNMM) makes up 4% [1,8,10], while sinonasal undifferentiated carcinoma (SNUC) and its subtypes represent 3–5% of cases [1,10]. Rarer histologies, such as sinonasal neuroendocrine carcinoma (SNEC), are less well studied [1,10]. Indeed, the sinonasal region presents the area of greatest histological diversity in the body, where tumors can and do arise from any and all tissues. The most common sites for sinonasal cancer vary depending on the histological subtype. For squamous-cell carcinoma (SNSCC), the most frequent site of origin is the maxillary sinus (~60%), followed by the nasal cavity (25%) and the ethmoidal complex (15%) [1]. The site of origin can influence symptoms, treatment outcomes, and overall quality of life [1].

Treatment is typically multimodal for most sinonasal malignancies, involving surgical resection where possible with perioperative chemotherapy and/or radiotherapy depending on histology and disease stage [1,11]. As these tumors arise near critical structures, treatment can often lead to diverse sequelae with substantial long-term morbidity that includes alterations to smell, the perception of flavor, vision, hearing, and cognition, in addition to the more general effects of systemic therapy. Standard follow-up involves the evaluation of adverse events in both the acute and delayed phases, which are typically graded using the Common Terminology Criteria for Adverse Events (CTCAE), where grade 1, 2, 3, and 4 events indicate mild, moderate, severe but not immediately life-threatening, and life-threatening events, respectively. Importantly, the standard of care varies for the different sinonasal malignancies, resulting in a heterogeneous landscape of long-term effects across the many distinct tumor types.

The National Coalition for Cancer Survivorship and American Cancer Society define survivorship as beginning at the time of diagnosis through the lifetime of the patient, where survivors include both the patient and their support network of friends, family, and caregivers [12,13]. The number of cancer survivors has increased substantially over the past two decades due to advancements in the management and treatment of malignancy. As such, many countries and healthcare systems have prioritized developing and integrating post-treatment plans to enable the provision of survivorship care, including through the establishment of specialist survivorship clinics. These clinics offer a valuable model for comprehensive, patient-centered care that could inform the development of care pathways for sinonasal malignancy survivors.

Following successful completion of treatment and disease control, sinonasal cancer survivors can transition from the multidisciplinary treatment team into a structured survivorship clinic to ensure continued support beyond just surveillance. Survivorship clinics—distinct from standard follow-up visits—are specifically designed to address the long-term and late effects of cancer and its treatment, including physical, functional, psychological, and social consequences. These clinics offer a more comprehensive, patient-centered model of care, incorporating survivorship care plans, patient-reported outcome measures, and the expertise of a broad multidisciplinary team, including psychologists/psychiatrists, oncologists, surgeons, speech and language therapists, nutritionists, dental care providers and rehabilitation specialists. In sinonasal cancer populations, survivorship clinics may enable earlier identification and management of issues such as sinonasal dysfunction, pain, nutrition, dental rehabilitation, social/financial issues, and psychosocial burden, which may otherwise be overlooked.

Several models of survivorship care exist, including primary care-led, multidisciplinary, and shared care approaches. The shared care model—in which oncology/surgical providers, primary care physicians, and allied specialists collaboratively manage survivorship—is increasingly recognized as both practical and effective, especially in complex cases. Survivorship clinics can serve as coordinating hubs within this model, helping to bridge care across hospital and community settings. However, the design and implementation of such clinics require careful consideration of several logistical factors: physical space constraints, which may be managed through telehealth and digital platforms; access to health professionals across key specialties (e.g., endocrinology, psychology, nutrition, social work, physical therapy, surgery); and appointment availability. The functionality and integration of secondary and primary care electronic medical records and digital communication tools are also critical to ensure continuity and coordination of care. Furthermore, provider education in survivorship care must be prioritized to standardize approaches and ensure quality. Where possible, survivorship clinics should also offer opportunities for research participation, helping to build much-needed evidence for best practices in this underrepresented population. While data specific to sinonasal cancer remains limited due to the rarity of the disease and the novelty of these care models, early institutional experience is promising. Future work should focus on evaluating outcomes and formalizing care pathways to support broader implementation and justify clinical investment [14,15,16,17].

Crucially, long-term and/or late effects occur in a substantial proportion of sinonasal cancer patients, affecting their everyday life, and recurrences can be common and occur late [18,19]. For head and neck cancer, several guidelines have been published regarding survivorship care [20,21,22,23]. However, these guidelines may not fully capture some of the more prominent issues associated with sinonasal malignancy specifically [10]. Indeed, due to the rarity of these tumors, there is limited evidence available in the published literature to guide their long-term management. Consequently, survivorship care is highly variable between centers. A lack of awareness of possible adverse effects, including late morbidity, which may arise months or years after treatment, may further exacerbate the long-term psychosocial well-being and quality of life of the survivor and may delay obtaining appropriate care.

**Figure 1:**
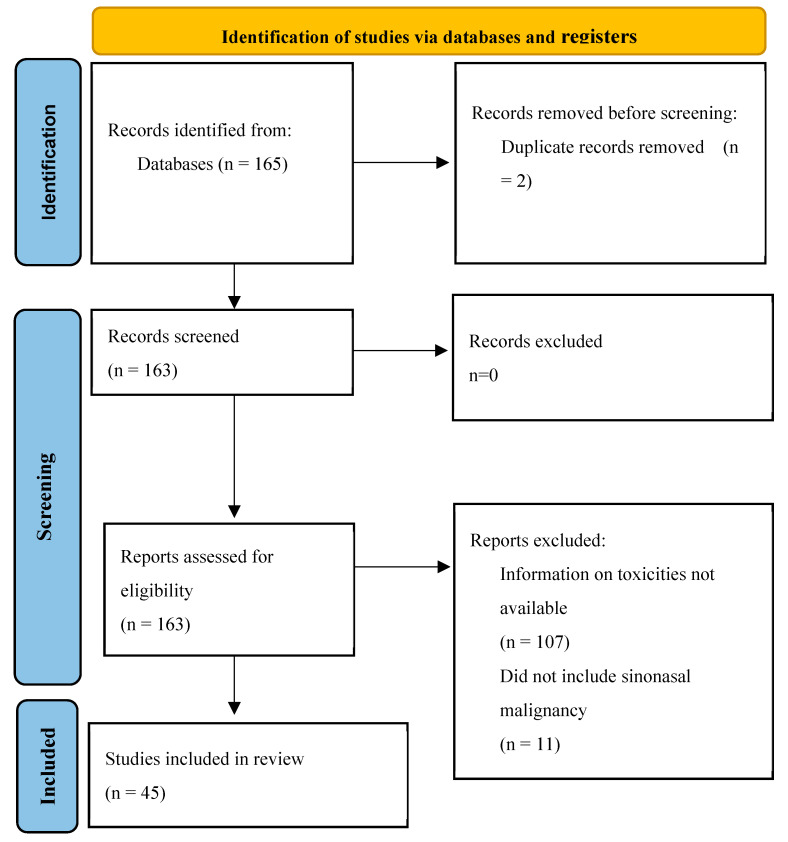
PRISMA Diagram.

This article provides an overview of the long-term and late effects of sinonasal cancers, based on a comprehensive literature review (Figure 1 and Table A1, with methods outlined in Appendix A). It aims to support patient education initiatives and highlight the diverse challenges faced by survivors. The long-term and late effects of sinonasal cancers, arising from the cancer itself or from its recurrence, progression, or treatment, are summarized in Table 1. Recognizing these effects is crucial for improving patient care, enabling the development of personalized treatment strategies, and ensuring the involvement of multi-disciplinary teams to address the complex needs of survivors.

## 2. Local Side Effects Related to the Sinonasal Region

Due to the aggressiveness of radical treatment with curative intent to the sinonasal region, which comprises surgery and adjuvant therapy, long-term sinonasal side effects occur in most patients. This might involve nasal discharge, persistent crusting, rhinitis and/or sinusitis, nasolacrimal duct obstruction, and alterations to sense of smell/perception of flavor either due to treatment or as a consequence of the underlying tumor, which can place a substantial burden on the patient’s quality of life [24,25,26,27,28,29].

The treatment of sinonasal cancers is multimodal and typically involves a combination of surgery (open, endoscopic, or both), radiotherapy, and chemotherapy. The extent of surgery depends on tumor location and size and may require maxillectomy with free flap reconstruction. These reconstructions can involve both soft tissue and bony components, with commonly used donor sites including the fibula, scapula, and iliac crest [19,29].

Radiation therapy may be delivered using conventional two-dimensional or three-dimensional conformal techniques or more advanced approaches such as intensity-modulated radiation therapy (IMRT) and particle therapy (e.g., proton therapy). IMRT, particularly at doses ≥60 Gy, has been associated with improved overall survival, decreased toxicities, and enhanced three-year recurrence-free survival in sinonasal squamous-cell carcinomas compared with conventional radiotherapy [1]. In larger or unresectable tumors, IMRT may also aid in preserving organ function while minimizing adverse effects.

Systemic chemotherapy typically involves platinum-based agents such as cisplatin, often in combination with other drugs [1]. Induction chemotherapy is frequently used for aggressive subtypes like sinonasal undifferentiated carcinoma (SNUC) and selected sinonasal squamous-cell carcinomas where definitive chemotherapy can be used for non-resectable tumors. Intra-arterial cisplatin with sodium thiosulfate has also been employed in prior studies for SNUC following radiotherapy [1].

For sinonasal mucosal melanoma immune checkpoint inhibitors have shown promise, particularly in cases of recurrent or persistent disease with or without distant metastasis. FDA-approved agents such as ipilimumab, pembrolizumab, and nivolumab have been used in the treatment of SNMM and are associated with prolonged survival [1,8].

The impact of surgery on sinonasal outcomes related to benign sinonasal issues, such as chronic rhinosinusitis, has been extensively studied. For sinonasal malignancy, outcomes are inherently distinct. Depending on the extent of tumor growth at presentation, some patients may undergo radical surgical approaches that may lead to substantial morbidity and disfigurement. Techniques such as maxillectomy with free flap reconstruction confer substantial morbidity, including anatomic airway obstruction from reconstruction and impairment of mucociliary function [29]. These can lead to symptoms including rhinorrhea, nasal obstruction, and facial pain or numbness. Furthermore, some patients may experience olfactory impairment (i.e., smell loss) due to surgical resection of the olfactory epithelium. The use of endoscopic techniques for piece-meal or en bloc resection with small tumors, when possible, has proven feasible and efficacious in preserving olfaction [30,31].

Importantly, over the past several decades, the safety and efficacy of an endoscopic approach for tumor resection has been demonstrated and is recommended where indicated. Recent studies have utilized the Sinonasal Outcome Test (SNOT)-20/22 patient-reported outcome measures (PROMs) to elucidate changes in sinonasal symptoms following primary treatment with an endoscopic approach [32]. In their study, Derousseau et al. [25] did not observe any improvements in the total SNOT-20 score and rhinologic subdomain scores two years post-endoscopic resection [25]. They did, however, observe improvements in the psychological and sleep domains. This contrasts with another study, in which a significant improvement in overall SNOT-22 score was observed over two years and across all subdomains [33]. However, in this study, a significant difference was observed between those who underwent adjuvant therapy and those who did not. While endoscopic approaches are associated with reduced postoperative morbidity and improved quality of life in some reports, these outcomes may partly reflect the earlier stage at which endoscopic surgery is often employed. However, data from Derousseau et al. demonstrate that more than half of the patients who underwent endoscopic resection had T4 tumors, indicating that this approach is increasingly used in advanced-stage disease as well and can also be considered as a palliative option for symptom control. Nonetheless, caution is warranted in interpreting outcome differences as direct evidence of surgical superiority, given the inherent risk of selection bias.

As treatment is often multimodal, patients often experience radiation-induced adverse effects as well. These may require further treatment, such as functional endoscopic sinus surgery for radiation-induced rhinosinusitis or operative debridement for nasal obstruction [34]. In a study of 27 patients treated for sinonasal malignancy with either primary or adjuvant intensity-modulated radiation therapy (IMRT), 17 patients experienced CTCAE grade 2 olfactory impairment, which seemed to be more prominent for those who underwent surgery and adjuvant IMRT, than those who underwent IMRT only [26]. Therefore, while outcomes improve with time post-treatment, depending on the course of treatment, there remains a subset of patients, who may continue to experience sinonasal symptoms for at least two years following primary treatment, which can adversely impact their quality of life.

## 3. Vision Impairments and Optic Nerve Toxicities

Long-term visual acuity impairment and other ophthalmologic morbidities may occur due to the proximity of the sinonasal region to the eye. Depending on the degree of orbital involvement, surgical resection ranging from orbital periosteum to orbital exenteration may be required with or without radiotherapy despite recent efforts to prioritize orbital preservation when possible [35].

Vision loss occurs in a subset of patients, who undergo radiotherapy, with lower rates observed for 3D compared to 2D radiotherapy [36,37]. In addition to vision loss, other symptoms include persistent tearing, which has been shown to occur in one-fifth of patients treated with IMRT [28]. Other conventional radiotherapy-induced sequelae include keratitis, cataracts, dry eye, and retinopathy [37,38]. Regarding proton-beam therapy, late adverse events include nasolacrimal stenosis, ectropion, conjunctivitis, blepharitis, dry eye, cataracts, and keratitis [39].

Regarding vision loss due to optic neuropathy, in their study of sinonasal cancers treated with primary or adjuvant IMRT, Sharma et al. [26] observed radiation-associated optic toxicity in 19% of patients, where the incidence of vision impairment correlated with the maximum received radiation dose [26,40]. This was similarly observed by Pala et al. [41], where severe late ocular toxicity was observed in 12% of patients, with permanent unilateral vision loss in a subset [41].

Other studies have focused on the impact of particle therapy (carbon ion or proton therapy), which is of particular interest given the recent inclusion of proton beam therapy as part of standard-of-care for the management of head and neck cancers in the United Kingdom. For head and neck tumors adjacent to the optic nerve, vision loss occurred between 17 and 58 months post-carbon ion or proton therapy in 11% of patients [42]. In a more recent cohort, CTCAE grade 3 or greater vision loss was observed in 5 of 143 nonmetastatic sinonasal cancer cases treated with primary or adjuvant proton therapy [43]. A total of 27.3% of 22 primary sphenoid carcinoma patients, most of whom presented with orbital invasion and were subsequently treated with definitive carbon ion therapy, demonstrated CTCAE grade 4 visual impairment after a median time of 47 months following treatment [44]. This was similar to another study where CTCAE grade 4 lateral visual loss was observed in 22.7% of patients treated with carbon ion therapy due to the proximity of the tumor to the optic nerve [45].

## 4. Ototoxicity

Damage to the inner ear is a common side effect of platinum-based chemotherapy, in particular, cisplatin, which can result in tinnitus and/or permanent sensorineural hearing loss. The prevalence of cisplatin-associated ototoxic hearing loss has been estimated to be 49.2% across all cancers, globally [46]. For head and neck cancers, including sinonasal malignancy, hearing loss has been observed in a substantial proportion of patients who received high-dose cisplatin (100 mg/m^2^) [47,48]. A lower-dose regimen comprising 40 mg/m^2^ weekly, which has been adopted more recently due to it having a more favorable toxicity profile, is associated with reduced prevalence of treatment-induced ototoxicity [47,49]. In a study of unresectable advanced-stage paranasal sinus carcinoma, ototoxicity (acute grade 3 or higher, per the Radiation Therapy Oncology Group radiation morbidity scoring criteria) occurred in three patients, who received concurrent chemoradiotherapy, with long-term hearing loss in two of these patients and serous otitis media in one [50]. Late ototoxicity was observed in two additional patients.

In addition to cisplatin-induced hearing loss, alterations to auditory structures from radiation, such as inflammation and fibrosis, can also lead to long-term ototoxicity in head and neck cancer patients [51]. In an early study of thirty-seven patients with paranasal sinus, nasal cavity, or lacrimal gland cancer, acute serous otitis media occurred in four patients within the first two weeks of radiotherapy and after treatment in three patients [52]. In a further two patients, hearing dysfunction occurred six months and four years following treatment, with otosclerosis due to radiotherapy in the former and bilateral hearing loss following induction chemotherapy and radiotherapy in the latter. In a more recent cohort of 112 patients treated surgically, the prevalence of middle ear effusion was 14% following treatment [53]. This prevalence was 35% for those with maxillary malignancy and was found to be a result of resection involving the nasopharynx, soft palate, eustachian tube, pterygopalatine fossa, and pharyngeal space.

## 5. Endocrinopathy

Damage to the pituitary gland can often be collaterally damaged in the treatment of head and neck cancers, where incidental irradiation can cause hormonal disturbances and hypopituitarism, with high radiation doses correlating with the rate of pituitary deficiency [54]. Snyers et al. [36] observed late hormonal disturbances (median follow-up 107 months) in 13 of 21 patients, with definitive hypopituitarism of multiple hormones in five patients [36]. The mean radiation dose to the pituitary gland and hypothalamus in these thirteen patients was significantly higher than in those who did not develop hormonal deficiencies. More recent studies further demonstrate late effects involving the pituitary, which can occur between one and six years following radiation treatment. In their study of 27 patients treated with primary or adjuvant IMRT, Sharma et al. [26] observed radiation-related hypopituitarism of at least one anterior pituitary hormone (CTCAE grade 1 or 2) in 6 patients, with a median time from first radiation fraction of 6.4 years [26]. Meanwhile, Contrera et al. [55] observed that, within their cohort of 50 patients treated with definitive or adjuvant radiation, of whom 30 had pituitary hormonal disturbances, there was a median time to diagnosis of pituitary dysfunction of 14 months [55]. Hyperprolactinemia was the most common abnormality with dysfunction of more than one axis observed in 13 patients.

## 6. Cognitive Changes and Brain Damage

Neurological and cognitive changes have been widely reported as long-term/late sequelae of head and neck cancer owing to the proximity of the treated area to critical brain structures. This involves radiation-induced damage to the brain, with reported cognitive changes to memory, concentration, and information processing, but may also include neurotoxicity associated with systemic therapy.

In their study of 27 sinonasal cancer patients treated with primary or adjuvant IMRT, Sharma et al. [56] observed cognitive impairment in 63% of the cohort, with a median of 6.4 years from the first radiation fraction to cognitive evaluation [56]. Furthermore, a statistically significant association was observed between radiation dose to specific brain structures and neurocognitive outcomes (e.g., impaired working memory and higher radiation dose to the right hippocampus, right temporal lobe, and right frontal lobe). Macroscopic abnormalities on MRI were observed in three patients, including radiation-induced necrosis, all of whom received maximum radiation doses above 60 Gy. In a separate, recent study, Yaniv et al. [57] observed declines in verbal learning and memory in 4% and 18% of patients, respectively, which may be associated with incidental irradiation to related brain structures [57].

Regarding proton therapy, treatment-induced neurological toxicities are limited, with most issues occurring in the acute phase and few long-term sequelae. CTCAE grade 3 or higher late central nervous system necrosis has been observed in 9 of 143 nonmetastatic sinonasal cancer cases treated with primary or adjuvant radiation [43]. This was similarly observed in a further cohort of 64 patients, in which only one acute CTCAE grade 3 physician-evaluated neurologic toxicity was observed and subsequently resolved, and furthermore in a study of 69 patients, in which no symptomatic brain necrosis was observed [58,59]. However, the risk of long-term toxicity is increased depending on the volume included in the irradiated region. For head and neck malignancies that require irradiation to the skull base, temporal lobe necrosis CTCAE grade 2 or higher has been observed in 5.6% of patients with 2-year temporal lobe and brain necrosis rates of 4.6% and 6.8%, respectively [60].

Conversely, radiation-induced brain injury is a common adverse effect of carbon ion therapy and can occur several years after initial treatment. In their study of 104 head and neck and skull base cancer patients, with a median follow-up of 45.5 months, Park et al. [61] observed CTCAE grade 2 or higher radiation-induced brain injury in 18% [61]. In a smaller cohort of twenty-two patients treated with definitive carbon ion therapy, Hagiwara et al. [44] observed CTCAE grade 3 or 4 brain necrosis in three patients, two of whom had presented with intracranial extension [44]. This was similar to a further study of twenty-two patients where five patients developed brain necrosis [45]. Lastly, in a study of 48 patients treated with proton therapy and a further 11 patients treated with carbon ion therapy, all of whom required irradiation of the brain parenchyma as part of primary treatment, Miyawaki et al. [62] observed brain alterations in 17% and 64%, respectively. Clinical symptoms, such as vertigo, headache, and epilepsy were observed in a subset [62]. Crucially, the authors found that the incidence of brain injury depended on both the radiation dose and volume included in high-dose regions.

## 7. Mastication and Swallowing Disorders

Treatment of more advanced sinonasal cancer may involve irradiation of other head and neck sites, including the cervical lymph nodes, with additional adverse effects including xerostomia (dry mouth), sore mouth due to infection and the formation of ulcers, dental caries, dysphagia (difficulty swallowing) and facial pain [34,63,64,65]. Importantly, a recent study demonstrated that patients with early disease experienced less morbidity than those who presented with more advanced disease, emphasizing the need for further efforts toward early detection [66].

Across head and neck cancers, xerostomia is the most frequently reported long-term effect, followed by dysphagia, which may be exacerbated by a history of smoking and the spread of the primary tumor to the lymph nodes [64,67]. In an earlier evaluation of long-term outcome for sinonasal cancers, Snyers et al. [36] observed mild xerostomia in 67% of patients [36]. Acute severe mucositis was further observed in 13 of 39 patients with unresectable advanced-stage paranasal sinus carcinoma [50]. A recent study of incompletely resected or inoperable sinonasal adenocarcinoma or squamous-cell carcinoma found that 12% of patients experienced acute severe mucositis; however, at two years post-treatment with combined IMRT and carbon ion boost, no long-term high-grade toxicities were observed [68]. Lastly, some patients may experience late osteoradionecrosis, which is a rare complication where damage to blood vessels during treatment can lead to bone death. In one study, this occurred 12 months or longer after initial treatment in 5 of 44 patients with sinonasal adenoid cystic carcinoma treated with radiotherapy [27].

Furthermore, in patients undergoing maxillectomy, the loss of teeth and alveolar bone on the affected side significantly impairs oral function, including the ability to chew and speak. The restoration of these structures, either through dentures, maxillary prostheses, or implants, is essential to improve daily dietary intake and prevent malnutrition [19]. Addressing oral dysfunction through prosthetic rehabilitation is crucial for improving nutritional status and overall well-being [19]. Furthermore, the incorporation of implants to support prosthetic obturators after maxillectomy has been shown to significantly improve oral function, chewing, and eating comfort [19]. This approach represents a viable and effective treatment modality for enhancing the functionality of prosthetic rehabilitation in maxillectomy patients, thus improving both their quality of life and long-term health outcomes. The challenges presented by maxillectomy in sinonasal cancer treatment underscore the need for comprehensive post-surgical care, including the restoration of oral structures to optimize recovery and minimize the risk of malnutrition and recurrence.

## 8. Quality of Life and Mental Health

The various physical alterations and impairments that persist and/or arise following treatment for sinonasal cancer place a substantial burden on the mental health, psychosocial well-being, and quality of life of the patient. The causes of these changes are multifactorial and can largely depend on the extent of the tumor at presentation, the subsequent treatment approach undertaken, and the ensuing long-term and/or late sequelae [69]. Furthermore, one recent study demonstrated a difference between disease-specific quality of life, which largely relates to symptoms associated with tumor burden, and generalized quality of life, which considers other treatment-related effects (e.g., that of chemotherapy/radiotherapy), where issues regarding the former tend to resolve in the acute phase following treatment, while the latter may take several years to resolve [69]. Tyler et al. [64] have further demonstrated that specific concerns related to treatment can predict the risk of reduced long-term quality of life [64].

Quality of life outcomes for patients who undergo endoscopic compared to open approaches have been widely studied for sinonasal malignancy and other benign processes, such as chronic rhinosinusitis. Importantly, endoscopic approaches are associated with reduced treatment-related morbidity and have been shown to significantly improve postoperative quality of life within the first year following treatment, particularly regarding sleep-related issues [25,70,71,72]. For advanced tumors, a more aggressive and invasive surgical approach may be required, which confers substantial morbidity, including consequent body image disturbance, which can have a substantial long-term effect on the patient’s psychosocial well-being [73,74]. Regarding patient-reported quality of life outcomes, Maoz et al. [69] observed worse generalized quality of life using the University of Washington Quality of Life Questionnaire (UWQOL) for patients who underwent an open surgical approach compared to endoscopic surgery [69]. In contrast, this difference was not observed by Deckard et al. [75] across various PROMs, including the Anterior Skull Base Questionnaire (ASBQ), MD Anderson Symptom Inventory (MDASI)-22, and SNOT-20 [75]. Similarly, Tyler et al. [64] did not observe statistically significant differences although a trend toward significance for improved ASBQ scores for patients treated exclusively with endoscopic surgery was noted [64]. Importantly, irrespective of surgical approach, the use of adjuvant chemotherapy and/or radiotherapy is associated with worse long-term quality of life [33,64,75].

In addition, due to the impact of treatment on olfaction and the physical function of the upper aerodigestive tract, some patients may be at risk of malnutrition [76]. Most patients will experience changes to their eating habits and their perception of food immediately after treatment, which may resolve within a few months. However, for many patients, these issues can persist for up to one year or longer. Not only does this impact the physical well-being of the patient but can also be extremely taxing both emotionally and psychologically.

A further important psychosocial aspect is the fear of recurrence, which is of relevance for sinonasal malignancy given the high rates of recurrence across histologies, with a risk of relapse of 12.2%, 23.5% and 30.5% at 1, 3, and 5 years, respectively. Importantly, recurrences may not be symptomatic, and for some histologies, such as olfactory neuroblastoma and adenoid cystic carcinoma, they can occur many years after the initial disease [77,78,79,80]. Recurrence after 5 years has been reported in 12% of cases of olfactory neuroblastoma [81]. As such, long-term surveillance is paramount but can further add to the burden on survivors’ psychosocial well-being and quality of life given the associated anxieties and stress. Crucially, one-third of sinonasal cancer patients will experience mental health illness due to their diagnosis [82]. At a median of 24 months post-treatment, 15% of patients had significant anxiety or depression, which was associated with advanced T-stage at diagnosis, as well as single status and having a worse social support system [83].

## 9. Financial Toxicity

Financial toxicity is the combined economic burden placed on the survivor (including both the patient and their caregiver(s)) by the direct cost of treatment, including that related to managing long-term sequelae, and indirect costs, such as lost productivity and wages. There is a lack of data on financial toxicity regarding sinonasal cancer specifically. However, several studies have evaluated this phenomenon across head and neck cancers, which have been summarized in a recent review [84]. In brief, studies have demonstrated a high prevalence of financial toxicity associated with head and neck cancer due to the high costs of therapy, which is typically trimodal, and the management of treatment-related adverse effects both in the acute and long-term/late phases, as well as the substantial impact of consequent disability on lifetime wages [84,85]. In certain cases, the costs associated with cancer care can persist beyond a decade after treatment [86]. Furthermore, various socioeconomic factors have been shown to predict worse financial toxicity, with low income, single status, and younger age conferring higher risk [85,87]. Crucially, financial toxicity has been shown to reduce post-treatment quality of life and oncologic outcomes [88,89,90,91]. Regarding lost productivity, this is of particular concern for head and neck cancer survivors due to the possibility of treatment-related visual/hearing and/or speech impairment, which can greatly impact the survivors’ day-to-day life, including the ability to engage in social activities and return to work following treatment.

Regarding sinonasal cancer, the long-term effects of treatment and the risk of late effects and recurrence many years after treatment can place a further financial strain on survivors. Prospective, longitudinal studies using validated approaches, such as the Comprehensive Score for financial Toxicity (COST) and the Financial Index of Toxicity (FIT) metrics, are much needed to better understand this under-appreciated toxicity [92,93]. Further study of risk factors related to financial toxicity in this context are also highly warranted in order to better identify those who may be especially impacted and provide support and mitigation strategies.

## 10. Patient Outreach and Initiatives

Given the extent and diversity of long-term and late effects experienced by survivors, it is essential for healthcare professionals to actively engage with patients and caregivers through patient and public involvement strategies. This includes addressing specific concerns and offering education via reading materials, online resources, and expert guidance. International guidelines stress the importance of clear communication about possible treatment effects and continued support for caregivers, family members, and partners as part of survivorship care. This may also involve establishing survivor groups and charities (e.g., the Head and Neck Cancer Research Trust, www.hncrtrust.org, accessed on 10 March 2025) to support community-based education. We hope this manuscript serves as a helpful resource for developing educational materials and guiding discussions at events like the Annual Olfactory Neuroblastoma Patient Education Meeting at Johns Hopkins University. However, there remains a critical lack of research on the physiological, psychosocial, and financial long-term effects of sinonasal cancer, highlighting the need for further research and improved multi-disciplinary care.

## 11. Conclusions

Sinonasal malignancies differ from other head and neck tumors due to their wide histological heterogeneity, leading to variability in prognosis, treatment, and functional outcomes. The anatomical complexity of the sinonasal region and its proximity to critical structures like the orbit and brain contribute to a wide range of treatment-induced adverse effects that may significantly affect long-term survivorship. Delayed morbidity and recurrence are also common, often appearing years after treatment. These long-term sequelae underscore the need for continuous care and support. As survival rates improve, survivorship clinics are becoming more common, yet further research is needed to better manage long-term and late effects and enhance survivors’ quality of life. A multi-disciplinary approach, supported by proper healthcare staff training, is essential to address these issues. Ongoing patient and public involvement will be key in identifying the most impactful challenges in survivors’ daily lives and informing future care strategies.

## Figures and Tables

**Table 1:** Summary of adverse effects associated with sinonasal cancers.

Adverse Effects Associated with Sinonasal Cancers	Description	Contributing Factors
Sinonasal Symptoms	Nasal discharge, persistent crusting, rhinitis, sinusitis, nasolacrimal duct obstruction, and facial pains/pressures. Loss of smell due to size or due to tumor resection or damage to olfactory epithelium.	Surgery (e.g., maxillectomy), radiation, tumor resection.Surgical resection of olfactory epithelium, radiation.
Vision Impairments	Vision loss, tearing, keratitis, cataracts, dry eye, retinopathy, ectropion, blepharitis, optic neuropathy. Radiation-associated optic toxicity, optic neuropathy leading to vision impairment.	Radiotherapy (IMRT, proton beam), surgery, proximity of tumor to optic nerve.
Ototoxicity	Tinnitus, sensorineural hearing loss, serous otitis media.	Cisplatin chemotherapy, radiation, surgery.
Endocrine Disorders	Hormonal disturbances, pituitary dysfunction, hypopituitarism.	Radiation dose to pituitary gland, surgical proximity.
Cognitive Impairment	Memory impairment, concentration issues, working memory deficits, neurotoxicity.	Radiation of the head and neck, chemotherapy.
Xerostomia	Dry mouth due to irradiation of salivary glands.	Radiation to head and neck area, mucositis secondary to chemotherapy.
Dysphagia	Difficulty swallowing.	Radiation, surgery near oropharynx, tumor location/size.
Quality of Life Impact	Decreased overall quality of life due to persistent physical symptoms.	Surgery, radiation, chemotherapy, fear of recurrence, treatment-related side effects.
Psychosocial Impact	Anxiety, depression, body image disturbance, fear of recurrence.	Tumor progression, surgical disfigurement, treatment side effects.
Financial Impact	Economic burden of treatment, loss of productivity.	High treatment costs in some nations, long-term care, lost wages due to disability from treatment-related impairments.

## Data Availability

No new data were created or analyzed in this study. Data sharing is not applicable to this article.

## Abbreviations

The following abbreviations are used in this manuscript:

ACC	Adenoid Cystic Carcinoma
ASBQ	Anterior Skull Base Questionnaire
COST	Comprehensive Score for Financial Toxicity
CTCAE	Common Terminology Criteria for Adverse Events
FIT	Financial Index of Toxicity
IMRT	Intensity-Modulated Radiation Therapy
ITAC	Intestinal-Type Adenocarcinoma
MDASI-22	MD Anderson Symptom Inventory
ONB	Olfactory Neuroblastoma
PROMs	Patient-Reported Outcome Measures
SNEC	Sinonasal Neuroendocrine Carcinoma
SNMM	Sinonasal Mucosal Melanoma
SNSCC	Squamous Cell Carcinoma
SNOT	Sinonasal Outcome Test
SNUC	Sinonasal Undifferentiated Carcinoma
UWQOL	University of Washington Quality of Life Questionnaire

## Appendix A

### Supplemental Methods

#### Supplemental Information

Systematic Literature Review Method

A systematic literature review was conducted to identify and synthesize evidence on long-term effects, survivorship outcomes, and late morbidity in patients treated for sinonasal cancer. The review adhered to PRISMA guidelines and aimed to provide a comprehensive overview of survivorship issues associated with this rare and heterogeneous group of malignancies.

The search strategy was collaboratively developed and independently executed by two reviewers (JL and UR) (Table A1). Searches were conducted across five electronic databases: PubMed/MEDLINE, Google Scholar, the Database of Abstracts of Reviews of Effects (DARE), DynaMed, and Embase. The first search was performed in December 2023 and the final search was performed on 24 April 2025, with no additional papers identified.

Search terms combined Medical Subject Headings (MeSH) and free-text keywords related to sinonasal neoplasms and survivorship concepts. Specifically, terms such as “Sinonasal Neoplasms”, “Paranasal Sinus Neoplasms”, “Skull Base Neoplasms”, “Survivors”, “Survivorship”, “Long-Term Effects”, “Late Effects”, and “Quality of Life” were used in various combinations with Boolean operators to ensure sensitivity and relevance of results. Filters were applied to limit studies to those published in English, conducted in human populations, and published between 1990 and 2025.

Following de-duplication, all titles and abstracts were screened for relevance. Articles were included if they reported on survivorship outcomes, treatment-related morbidity, long-term effects, or quality of life in patients with sinonasal cancer. Studies not focused on sinonasal malignancies, or those that lacked survivorship outcomes, were excluded. Full-text review was performed independently by the two reviewers. Any discrepancies in article inclusion were resolved through discussion with a third, independent reviewer (ML) until consensus was reached.

This methodology enabled the identification of studies that addressed the diversity and complexity of survivorship experiences in sinonasal cancer patients and supported the review’s aim of informing both clinical practice and future research priorities.

**Table A1:** Search strategy.

Category	Terms
Primary Terms	“Sinonasal Neoplasms” [MeSH] OR “Paranasal Sinus Neoplasms” [MeSH] OR “Skull Base Neoplasms” [MeSH]
Survivorship-Related Terms	“Survivors” [MeSH] OR “Survivorship” [MeSH] OR “Long-Term Effects” [MeSH] OR “Quality of Life” [MeSH] OR “Late Effects” [MeSH]
Boolean Combination	(“Sinonasal Neoplasms” [MeSH] OR “Paranasal Sinus Neoplasms” [MeSH] OR “Skull Base Neoplasms” [MeSH]) AND (“Survivors” [MeSH] OR “Survivorship” [MeSH] OR “Long-Term Effects” [MeSH] OR “Quality of Life” [MeSH] OR “Late Effects” [MeSH])
Filters Applied	Language: English [Filter] Population: Humans [Filter] Publication Date: 1990–2025
Date of Search	24 April 2025
Databases Searched	PubMed, Google Scholar, DARE, DynaMed, Embase
Articles Retrieved	175
